# Emergency Department Crowding: Time for Interventions and Policy Evaluations

**DOI:** 10.1155/2012/838610

**Published:** 2012-02-07

**Authors:** Adrian Boyle, Kathleen Beniuk, Ian Higginson, Paul Atkinson

**Affiliations:** ^1^Emergency Department, Cambridge University Foundation Hospitals NHS Trust, Hills Road, Cambridge CB2 2QQ, UK; ^2^Engineering Design Centre, Cambridge University, Cambridge CB2 1PZ, UK; ^3^Emergency Department, Plymouth Hospitals NHS Trust, Derriford Road, Crownhill, Plymouth, Devon PL6 8DH, UK; ^4^Emergency Department, St John Regional Hospital, New Brunswick, Canada

## Abstract

This paper summarises the consequences of emergency department crowding. It provides a comparison of the scales used to measure emergency department crowding. We discuss the multiple causes of crowding and present an up-to-date literature review of the interventions that reduce the adverse consequences of crowding. We consider interventions at the level of an individual hospital and a policy level.

## 1. Introduction

Emergency department crowding is one of the leading problems facing emergency physicians, nurses, and their patients, in most developed countries. It has been proposed that emergency department crowding is the equilibrium state of the current health care system [1]. While this may be so, it is not safe; there is a large body of evidence that patients are harmed in crowded emergency departments [2]. Crowded departments threaten delivery of timely care. Delays to analgesia, antibiotic therapy, and thrombolysis or percutaneous coronary intervention are all well described [2–6]. Compliance with other recognised care standards is reduced. Regular medications are omitted in elderly frail patients. One author has estimated that more people die avoidably as the result of crowding in New Zealand than in road traffic collisions [7]. Similar opinions have been expressed by Australian authors, though the weakness of the underlying evidence is acknowledged [8]. Patients with more complex needs are more likely to board in the emergency department. Studies have shown that frail, elderly patients and critically ill patients are more likely to spend disproportionate time boarding in the emergency department. Crowding also impairs dignity, privacy, and completeness of care.

A crowded emergency department creates problems beyond that department. Ambulance crews are unable to unload their patient. This reduces resilience and the capacity of prehospital services to respond to calls [9].

Patients harmed by crowding in an emergency department continue to suffer after they have been admitted. There is some evidence that patients admitted through crowded emergency departments have longer hospital stays [10]. Emergency patients are also more likely to be admitted when the emergency department is crowded. This is most likely because the emergency department's ability to safely discharge patients is compromised.

Crowding also harms staff. There are associations with absenteeism, staff sickness, and burnout. This results in experienced staff leaving and more junior staff, or agency staff delivering an increasingly busy and inefficient service. Resident and student education is compromised [11, 12]. Recruitment is harmed.

## 2. Definition of Crowding

Despite a large literature describing the consequences of crowding, there is little consensus on a definition for crowding. The term “overcrowding” should be abandoned, as *any *crowding is harmful. Defining crowding is important, as it allows measurement, subsequent research, and policy evaluation. There are other measures; simple bed occupancy has face validity and compares moderately to other scales [13]. Simply counting the number of patients who leave before treatment is simple, but ignores the complexity of crowding [14]

There are a number of crowding scales in the literature, though many are limited by being country specific or lack a gold standard in development and are incompletely validated, see Table 1. These mainly aim to quantify crowding. Our research group (KB and AB) have developed an eight point operational definition, and we are working to validate this measure, see Table 2.

There are four other scales in the literature, all of which perform moderately with clinician's perceptions of crowding [15].

## 3. Causes of Crowding

**Figure 1:**
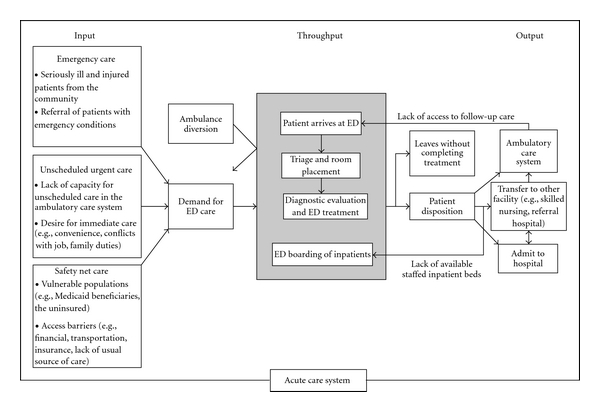
Asplin's model of acute care.

Crowding is caused by multiple factors. These can be best thought of in terms of input, throughput, and output. Asplin's conceptual model illustrates the stages that can lead to emergency department crowding [16] see Figure 1.

Input factors include not only the volume, but also the acuity and type of patients. Worldwide the volume of patients attending emergency departments has increased dramatically over the last 20 years [17]. The reasons for this are not well understood. Primary care has also seen a substantial increase in activity in the same time period. Older people, a growing group, typically require disproportionate care [18]. Patients with mental illness and critical care patients require extensive emergency department care. A small increase in any of these groups has a knock-on effect. “Inappropriate attenders,” a judgemental term for patients who could receive medical care elsewhere, do not significantly contribute to crowding. Input problems need not cause crowding if the rest of the emergency admission and discharge process works well.

Throughput factors refer to activities within the emergency department that can hinder patient flow. Emergency departments are extremely complex systems and almost any activity can lead to crowding. Poor emergency department design, which does not support flow, contributes to crowding. A linearly designed emergency department, where cubicles flank a long straight corridor, is probably most efficient. Having adequate physical space helps. However, merely increasing cubicle spaces does not reduce crowding if processes within the department and in the main hospital are not improved [4]. Delays with diagnostic imaging and laboratory results may contribute to crowding. Inadequate numbers of medical and nursing staff may also be a factor. Increasingly stringent care standards for conditions such as sepsis, transient ischaemic attack and stroke have increased the workload of emergency departments. Patient and professional expectations are higher. Analysis of the separate components of the time patients spend in the emergency department has shown that waiting comprises 51–63% of total patient turnaround time. Major components are time away for radiological investigations, waiting time for the first physician's examination, and waiting time for blood work [19]. Output factors are the main cause of emergency department crowding [20, 21]. Lack of inpatient beds is the single most important cause of crowding. A lack of critical care beds leads to high acuity patients remaining in the emergency department. Worldwide the trend has been to reduce inpatient bed capacity. Quality standards such as single sex compliance in the NHS, and infection control policies, have further contributed, though they are difficult to quantify. There is a potential for harm in that patients transferred as outliers on other wards have longer stays and more harm this in turn reduces hospital capacity and drives further crowding. There is a strong perception that hospitals prioritise more lucrative elective work over emergency admissions.

## 4. Interventions

There is an extensive literature about the harms of emergency department crowding, and a smaller literature about effective interventions [2, 22]. Interventions can also be grouped into input, throughput, and output. The strongest evidence comes from throughput solutions. This is a paradox, as these have the least effect on crowding, as the main cause is usually access block [16]. Before interventions are instituted, it is critical to identify what the main causes of crowding are in an individual emergency department. Failure to take this logical step leads to effort being expended on unnecessary interventions.

Reducing inputs is attractive to administrators and policy makers. The evidence of effect is very weak. Administrators often focus on inappropriate attenders. The best estimate is that 10–15% of patients attending emergency departments in the British NHS could have been treated adequately by a general practitioner [23]. This is considerably less than the estimate used by many policy makers. Effort expanded on directing these patients away from the emergency department once they have arrived is rarely worthwhile [24]. Many patients have tried to access primary care beforehand [25]. Developing alternative sources of care away from emergency departments, such as walk-in centres, has probably met a previously unmet need [26]. Colocation of primary care services within or adjacent to emergency care services is helpful, but the evidence is weak and the cost effectiveness is uncertain [23]. Diverting ambulance patients to other hospitals is rarely an option in a few of the major British urban centres, though it is more widely practised in the rest of the Western world. It does not ease crowding, rather then being a response to it. A patient admitted to distant hospital may suffer a longer stay and repeated, unnecessary investigations.

There are a variety of throughput interventions to reduce crowding in emergency departments. Ensuring patients are seen early by a senior emergency physician who can “front-load” investigations is helpful [27]. However, this is arduous and tying up a senior emergency physician at the front door has costs elsewhere in the emergency department. Training nursing staff to order X-rays at triage is helpful and cuts the patient's stay by around 20 minutes [28]. Ensuring that staff are multiskilled also helps so that there are less bottlenecks. However, these interventions help with lower acuity patients, but not high acuity patients. For instance, ensure that as many staff are able to perform routine phlebotomy and place plasters as possible. “Streaming,” by which patients are grouped into broad acuity categories and managed through separate processes, reduces overall waiting times. Streaming is unlikely to be helpful if the main cause of crowding is inadequate hospital capacity. Monitoring key bottlenecks, such as time for laboratory and radiology results to become available, is useful. Streamlining transfer policies, so that low acuity patients can be transferred to inpatient wards by portering staff alone, supported by a telephone handover is not proven, but seems sensible. Developing ambulatory care pathways to avoid admission for patients with defined conditions, such as transient ischaemic attacks, cellulitis, deep vein thrombosis, and pulmonary embolism makes clinical sense, but requires investment. Identifying investigative pathways, such as point-of-care testing, that reduce the amount of time a patient spends in the hospital, is attractive [29]. However, these should be restricted to patient groups where the evidence base indicates that this is as safe as standard care.

Sending well patients home to await results that are anticipated to be normal is helpful, provided the patient is able to return to the hospital and the clinician is able to contact the patient. This is less helpful where the patient lives a significant distance from the hospital.

National targets, such as the four-hour standard in the British NHS, are controversial [17]. The UK standard, which requires 95% of all emergency patients to been admitted or discharged from the emergency department within four hours, have been associated with an increase in attendances, though this may not be causal. This may drive crowding. There are concerns that the standard is set too high and distorts clinical priorities [30, 31].

## 5. Output Solutions

Individual hospitals need to have full capacity protocols, with agreed and defined triggers. These protocols recruit support from in-patient services, focus the minds of bed managers and set clearly defined thresholds and actions. These need to be developed locally and take account of local resources. Many hospitals struggle to have enough capacity to deal with surges in emergency department activity. Flexible scheduling models for emergency department medical and nursing staff have been proposed, but often these pose problems with job satisfaction and complicate personal commitments. Emergency physicians and their administrators face an uphill struggle to engage administrators and clinicians elsewhere in the hospital to assist with emergency department crowding. Prompt discharging of patients from wards can be difficult, particularly when patients require medication to be dispensed from a pharmacy, or specialised transport services. Discharge lounges, where discharged patients can wait before transfer, help reduce hospital capacity. Early ward rounds of newly admitted patients help to match bed availability with demand. Boarding patients on inpatient wards, where a patient is sent to a full ward, to await a bed, is controversial [32, 33]. While there is a wealth of evidence that patients come to harm in crowded emergency departments, we were unable to find evidence that boarded patients come to harm on inpatient wards. This lack of evidence probably reflects that fact that the studies have not been done, rather than absence of effect. Despite this, professional bodies have consistently pragmatically endorsed boarding on inpatient wards [32, 34]. Moving only a few boarded patients from a crowded emergency department has a minimal effect on inpatient wards but has a marked and beneficial effect on the emergency department.

## 6. Conclusions

Can we afford to continue with the current state of emergency department crowding? Will the current equilibrium shift? Is there perhaps an administrative acceptance that there will always be a queue for acute care and that the emergency department is where that will be?

Policy makers and commissioners of emergency services need to consider emergency department crowding as an unintended consequence of policies and consider how they can incentivise the whole emergency healthcare system to function effectively.

Emergency department crowding is an increasingly recognised problem across the world. While the evidence is clear of the harms, future work needs to systematically evaluate interventions and guide evidence-based policy.

## Figures and Tables

**Table 1:** A comparison of crowding scales.

Crowding Scale	Calculation	Outcome	Notes
Real-time emergency analysis of demand Indicators scores (READI) [35]	DV = (BR + PR) × AR	DV > 7 indicates overcrowding, and further assessment is recommended.	The acuity ratio is based on a four-level acuity scale (4 being most acute).
	$\text{BR}=\frac{\left(\text{total}\;\text{ED}\;\text{patients}+\text{predicted}\;\text{arrivals}-\text{predicted}\;\text{departures}\right)}{\text{ED}\;\text{spaces}}$	A BR > 1 indicates overcrowding.	Poor agreement between READI score and staff perception of crowding [36].
	$\text{PR}=\frac{\text{arrival}\;\text{sper}\;\text{hour}}{\sum \text{PPH}\;\text{for}\;\text{each}\;\text{physician}}$	A PR > 1.5 indicates an understaffed ED.	
	$\text{AR}=\frac{\sum \left(\text{triage}\;\text{category}\right)\left(\text{patients}\;\text{at}\;\text{each}\;\text{category}\right)}{\text{number}\;\text{of}\;\text{patients}}$	AR close to 1 indicates a low burden of illness: AR close to 4 indicates a severe burden of illness.	
	Demand value (DV) provides an overall measure of demand based on current calculations of the three ratios.		
	Bed ratio (BR) assesses the demand per treatment space.		
	Provider ratio (PR) calculates how many patients can be seen by the physician providers based on the average number of patients seen per hour		
	(PPH) by each physician.		
	Acuity ratio (AR) measures the relative burden of illness by averaging the triage categories of all patients in the ED.		

Emergency department work index (EDWIN) [37]	$\text{EDWIN}=\frac{\sum n_{i}{\text{t}}_{i}}{N_{a}\left(B_{T}-B_{A}\right)}$ The number of patients present in the ED in triage category *i*. The triage category (ordinal scale 1–5, 5 being most acute). The number of attending physicians on duty at a given time. The total number of beds, or treatment bays, available in the ED. The number of admitted patients (boarders) in the ED.	EDWIN score < 1.5 → Active but manageable ED EDWIN score 1.5–2.0 → A busy ED has an EDWIN score > 2 → A crowded ED	The triage system used was an inverted emergency severity index (ESI). The scale was initially evaluated against nurse/physician perception of crowding. Has been shown to be a strong predictor of ambulance diversion and to correlate well with staff perception of crowding [38].

Emergency department crowding score	Exact calculations for the EDCS are unclear, however, the specific inputs to the EDCS are the following: (i) number of attending emergency physicians, (ii) number of staffed ED beds, (iii) number of critical care patients, (iv) number of total ED patients, (v) number of staffed hospital beds, (vi) hospital occupancy rate.	EDCS score > 65 was found to be predictive of both ambulance diversion and the number of patients who leave without being seen by a physician.	Generates an output score between 0 and 100. Three variables were identified that independently predicted physician and nurse ratings of EDC: (1) the number of boarders, (2) the total number of ED patients, and (3) the number of critical care ED patients.

National emergency department overcrowding study (NEDOCS) [26]	$\begin{array}{cc} \text{NEDOCS} & =-20+85.8\times \left(\frac{\text{Total}\;\text{patients}}{\text{ED}\;\text{Beds}}\right)\\ & \;+600\times \left(\frac{\text{admits}}{\text{hospital}\;\text{beds}}\right)\\ & \;+13.4\times \left(\text{ventilators}\right)+0.93\times \left(\text{longest}\;\text{admit}\right)\\ & \;+5.64\times (\text{Last}\;\text{bed}\;\text{time}) \end{array}$	0–50: normal 51–100: busy 101–140: overcrowded 141–180: severe >180: disaster	Generates an output score between 0 and 200 however higher scores are possible. Weiss has demonstrated that NEDOCS is highly correlated with clinicians' perceptions of crowding, ambulance diversion, and patients leaving without being seen [39]

	Total Patients	Total number of patients in the ED, including those in the waiting room, fast track	
		or observation areas.	
	ED Beds	Total number of ED beds, including those in hallways, fast track areas, chairs,	
		and elsewhere.	
	Admits	Total number of boarders/admitted patients in the ED at the time the score is	
		calculated.	
	Hospital Beds	Total number of hospital beds, typically the number of licensed beds that could	
		be used in a disaster.	
	Ventilators	The number of patients in the ED on ventilators or respirators	
	Longest Admit	The longest patient boarding time (in hours) at which the score is calculated	
	Last Bed Time	The time (in hours) from arrival to bed for the last patient assigned to a bed.	

ED work Score [40]	$\text{Work}\;\text{score}=3.23\times \left(\frac{P_{\text{wait}}}{B_{T}}\right)\;+0.097\times \left[\frac{\sum n_{i}t_{i}}{N_{n}}\right]+10.92\times \left(\frac{B_{A}}{B_{T}}\right)$	Using the threshold work score = 4.77, predict the decision for ambulance diversion with 86% sensitivity and 80% specificity.	Published in 2006 by Epstein and Tian Developed to be used in real time to direct ambulance traffic based on an objective measure of ED status. The triage system used was an inverted emergency severity index (ESI). Sometimes referred to as the Boston ED workscore.

	*P*_wait_	Number of patients in the waiting room.	
	*B*_*T*_	The total number of beds, or treatment bays, available in the ED.	
	*n*_*i*_	The number of patients present in the ED in triage category *i*.	
	*t*_*i*_	The triage category (ordinal scale 1–5, 5 being most acute).	
	*N*_*n*_	Number of nurses on duty.	
	*B*_*A*_	The number of admitted patients (boarders) in the ED.	

ED occupancy Rate	$\text{Occupancy}\;\text{rate}=\left(\frac{\text{total}\;\text{patients}}{B_{T}}\right)$	ED occupancy rate above 1.0 indicates there are more ED patients than treatment bays. The threshold to indicate crowding differs from study to study. Examples are OR = 1.0, 1.2, and 1.4.	Suggested to be the simplest and overall best indicator of crowding. [13, 41]

	Total patients (number of patients in the ED including those in the waiting room, boarding, hallway, and chairs.)		
	BT (the total number of licensed treatment bays including fast track or observation units, excluding hallway locations.)		

**Table 2:** A consensus definition of emergency department crowding [42].

Input measures	
(1) Ability of ambulances to offload	
An ED is crowded when the 90th percentile time between ambulance arrival and offload is greater than 15 minutes.	
(2) Patients who leave without being seen or treated (LWBS)	
An ED is crowded when the number of patients who LWBS is greater than or equal to 5%.	
(3) Time until triage	
An ED is crowded when there is a delay greater than 5 minutes from the time of patient arrival to the begining of their initial triage.	

Throughput measures	

(4) ED occupancy rate	
An occupancy rate is the total volume of patients in the ED compared to the total number of officially designated ED treatment	
spaces. An ED is crowded when the occupancy rate is greater than 100%.	
(5) Patients' total length of stay in the ED	
An ED is crowded when the 90th percentile patient's; total length of stay is greater than 4 hours.	
(6) Time until a physician first sees the patient	
An ED is crowded when an emergent patient waits longer than 30 minutes to be seen by a physician.	

Output measures	

(7) ED boarding time	
An ED is crowded when less than 90% of patients have left the ED 2 hour after the admission decision.	
(8) Number of patients boarding in the ED	
Boarders are defined as admitted patients waiting to be placed in an inpatient bed. An ED is crowded when there is greater than	
10% occupancy of boarders in the ED.	

## References

[1] McCain R, Hamilton R, Linnehan F, Osinga S, Hofstede G, Verwaart T (2011). Emergency department overcrowding as a Nash equilibrium: hypothesis and test by survey methodology. *Lecture Notes in Economics and Mathematical Systems*.

[2] Morris ZS, Boyle A, Beniuk K, Robinson S Emergency department crowding: towards an agenda for evidence-based intervention.

[3] Schull MJ, Vermeulen M, Slaughter G, Morrison L, Daly P (2004). Emergency department crowding and thrombolysis delays in acute myocardial infarction. *Annals of Emergency Medicine*.

[4] Hwang U, Richardson LD, Sonuyi TO, Morrison RS (2006). The effect of emergency department crowding on the management of pain in older adults with hip fracture. *Journal of the American Geriatrics Society*.

[5] Kulstad EB, Kelley KM (2009). Overcrowding is associated with delays in percutaneous coronary intervention for acute myocardial infarction. *International Journal of Emergency Medicine*.

[6] Moskop JC, Sklar DP, Geiderman JM, Schears RM, Bookman KJ (2009). Emergency department crowding, part 1-concept, causes, and moral consequences. *Annals of Emergency Medicine*.

[7] Johnston M (2008). *Hundreds Die Because of Hospital Crowding*.

[8] Forero R, Hillman KM, McCarthy S, Fatovich DM, Joseph AP, Richardson DB (2011). Access block and emergency department overcrowding. *Critical Care*.

[9] Eckstein M, Chan LS (2004). The effect of emergency department crowding on paramedic ambulance availability. *Annals of Emergency Medicine*.

[10] Hoot NR, Aronsky D (2008). Systematic review of emergency department crowding: causes, effects, and solutions. *Annals of Emergency Medicine*.

[11] Atzema C, Bandiera G, Schull MJ, Coon TP, Milling TJ (2005). Emergency department crowding: the effect on resident education. *Annals of Emergency Medicine*.

[12] Jelinek GA, Weiland TJ, MacKinlay C (2010). Supervision and feedback for junior medical staff in Australian emergency departments: findings from the emergency medicine capacity assessment study. *BMC Medical Education*.

[13] McCarthy ML, Aronsky D, Jones ID (2008). The emergency department occupancy rate: a simple measure of emergency department crowding?. *Annals of Emergency Medicine*.

[14] Pines JM (2006). The left-without-being-seen rate: an imperfect measure of emergency department crowding. *Academic Emergency Medicine*.

[15] Jones SS, Allen TL, Flottemesch TJ, Welch SJ (2006). An independent evaluation of four quantitative emergency department crowding scales. *Academic Emergency Medicine*.

[16] Asplin BR, Magid DJ, Rhodes KV, Solberg LI, Lurie N, Camargo CA (2003). A conceptual model of emergency department crowding. *Annals of Emergency Medicine*.

[17] Thompson C, Hayhurst C, Boyle A (2010). How have changes to out-of-hours primary care services since 2004 affected emergency department attendances at a UK district general hospital? a longitudinal study. *Emergency Medicine Journal*.

[18] George G, Jell C, Todd BS (2006). Effect of population ageing on emergency department speed and efficiency: a historical perspective from a district general hospital in the UK. *Emergency Medicine Journal*.

[19] Sinreich D, Marmor Y (2005). Ways to reduce patient turnaround time and improve service quality in emergency departments. *Journal of Health, Organisation and Management*.

[20] Fatovich DM, Hirsch RL (2003). Entry overload, emergency department overcrowding, and ambulance bypass. *Emergency Medicine Journal*.

[21] Fatovich DM, Nagree Y, Sprivulis P (2005). Access block causes emergency department overcrowding and ambulance diversion in Perth, Western Australia. *Emergency Medicine Journal*.

[22] Moskop JC, Sklar DP, Geiderman JM, Schears RM, Bookman KJ (2009). Emergency department crowding, part 2—barriers to reform and strategies to overcome them. *Annals of Emergency Medicine*.

[23] Carson D, Clay H, Stern R (2010). Primary care and emergency departments.

[24] Derlet RW, Kinser D, Ray L, Hamilton B, McKenzie J (1995). Prospective identification and triage of nonemergency patients out of an emergency department: a 5-year study. *Annals of Emergency Medicine*.

[25] Benger JR, Jones V (2008). Why are we here? a study of patient actions prior to emergency hospital admission. *Emergency Medicine Journal*.

[26] Salisbury C, Munro J (2003). Walk-in centres in primary care: a review of the international literature. *British Journal of General Practice*.

[27] Rowe BH, Guo X, Villa-Roel C (2011). The role of triage liaison physicians on mitigating overcrowding in emergency departments: a systematic review. *Academic Emergency Medicine*.

[28] Lindley-Jones M, Finlayson BJ (2000). Triage nurse requested x rays—are they worthwhile?. *Journal of Accident and Emergency Medicine*.

[29] Goodacre S, Bradburn M, Fitzgerald P (2011). The RATPAC (randomised assessment of treatment using panel assay of cardiac markers) trial: a randomized controlled trial of point-of-care cardiac markers in the emergency department. *Health Technology Assessment*.

[30] Mason S, Nicholl J, Locker T (2010). Four hour emergency target. Targets still lead care in emergency departments. *British Medical Journal*.

[31] Locker T, Mason S, Wardrope J, Walters S (2005). Targets and moving goal posts: changes in waiting times in a UK emergency department. *Emergency Medicine Journal*.

[32] American College of Emergency Physicians (2011). Boarding of admitted and intensive care patients in the emergency department. *Annals of Emergency Medicine*.

[33] Walsh P, Cortez V, Bhakta H (2008). Patients would prefer ward to emergency department boarding while awaiting an inpatient bed. *Journal of Emergency Medicine*.

[34] (2006). Emergency nurses association position statement: crowding in the emergency department. *Journal of Emergency Nursing*.

[35] Reeder TJ, Garrison HG (2001). When the safety net is unsafe: real-time assessment of the overcrowded emergency department. *Academic Emergency Medicine*.

[36] Reeder TJ, Burleson DL, Garrison HG (2003). The overcrowded emergency department: a comparison of staff perceptions. *Academic Emergency Medicine*.

[37] Bernstein SL, Verghese V, Leung W, Lunney AT, Perez I (2003). Development and validation of a new index to measure emergency department crowding. *Academic Emergency Medicine*.

[38] Jones SS, Allen TL, Flottemesch TJ, Welch SJ (2006). An independent evaluation of four quantitative emergency department crowding scales. *Academic Emergency Medicine*.

[39] Weiss SJ, Ernst AA, Nick TG (2006). Comparison of the national emergency department overcrowding scale and the emergency department work Index for quantifying emergency department crowding. *Academic Emergency Medicine*.

[40] Epstein SK, Tian L (2006). Development of an emergency department work score to predict ambulance diversion. *Academic Emergency Medicine*.

[41] Hoot NR, Zhou C, Jones I, Aronsky D (2007). Measuring and forecasting emergency department crowding in real time. *Annals of Emergency Medicine*.

[42] Beniuk K, Boyle A, Clarkson J Emergency department crowding: developing an operational definition using a Delphi study.

